# Role of Prion Protein Aggregation in Neurotoxicity

**DOI:** 10.3390/ijms13078648

**Published:** 2012-07-11

**Authors:** Alessandro Corsaro, Stefano Thellung, Valentina Villa, Mario Nizzari, Tullio Florio

**Affiliations:** Section of Pharmacology, Department of Internal Medicine, University of Genova, Genova 16132, Italy; E-Mails: 606015@unige.it (A.C.); stefano.thellung@unige.it (S.T.); valentina.villa@unige.it (V.V.); mario.nizzari@unige.it (M.N.)

**Keywords:** transmissible spongiform encephalopathies, prion protein aggregation, hPrP90-231 mutants and *wild type*, cell internalization

## Abstract

In several neurodegenerative diseases, such as Parkinson, Alzheimer’s, Huntington, and prion diseases, the deposition of aggregated misfolded proteins is believed to be responsible for the neurotoxicity that characterizes these diseases. Prion protein (PrP), the protein responsible of prion diseases, has been deeply studied for the peculiar feature of its misfolded oligomers that are able to propagate within affected brains, inducing the conversion of the natively folded PrP into the pathological conformation. In this review, we summarize the available experimental evidence concerning the relationship between aggregation status of misfolded PrP and neuronal death in the course of prion diseases. In particular, we describe the main findings resulting from the use of different synthetic (mainly PrP106-126) and recombinant PrP-derived peptides, as far as mechanisms of aggregation and amyloid formation, and how these different spatial conformations can affect neuronal death. In particular, most data support the involvement of non-fibrillar oligomers rather than actual amyloid fibers as the determinant of neuronal death.

## 1. Introduction

Although most proteins evolved to efficiently fold into a unique native structure, misfolding occurs frequently *in vivo* [1]. Alteration of protein conformation is a common neuropathological denominator for most neurodegenerative disorders, including Parkinson, Alzheimer’s and Huntington diseases, as well as transmissible spongiform encephalopathies (TSE, or prion disease) [2]. Each of these disorders is characterized by misfolding of specific proteins, *i.e.*, α-synuclein in Parkinson, β-amyloid and tau in Alzheimer’s, huntingtin in Huntington diseases, and prion protein (PrP) in TSE [3]. Misfolded proteins transform into aggregation pathways via β-pleated sheets and this process is believed to be responsible of the neurodegeneration observed in these diseases. When incorrectly folded, all these pathogenic proteins escape intracellular proteolytic turnover, acquiring the propensity to aggregate and accumulate inside neurons, suggesting that alterations in protein homeostasis are general pathogenic features of otherwise clinically and etiologically distinct diseases [4].

In the late 1950s, an amyloid structural model was proposed by Cohen and Calkins, which first reported its non-branching fibrillar structure, as observed by electron microscopy [5].

In 1968, Eanes and Glenner showed by X-ray diffraction amyloid to be β-sheet rich with the β strands perpendicular to the long axis of the fibrils, representing the distinctive “cross-β” pattern [6]. This evidence brought to light that amyloids are ordered filamentous structures, and not simply amorphous deposits in tissue.

Currently, amyloid is defined as highly-ordered protein aggregates, with a filamentous morphology of indefinite length, and diameters of approximately 2–20 nm. Amyloid fibrils are rich in β-sheet secondary structure and formed by non-covalent polymerization of a single protein with the polypeptide chains aligned in the cross-β configuration [7]. The original belief that only a limited number of proteins can aggregate in amyloid structures has been recently disproved by the evidence that almost all proteins have the structural characteristics responsible for amyloidogenesis [8,9]. The amyloid aggregation process proceeds through several organization states, including *β* sheet-rich dimers, trimers, tetramers, low molecular weight prefibrillar oligomers, protofibrils, to reach the final insoluble fibrillar structure [10].

Although protein aggregation was soon considered a critical process for neurodegenerative diseases, only at the end of the 1990s were amyloid aggregates identified as responsible for neuronal death [11]. Today there is a general agreement that the pathogenic forms produced in neurodegenerative conditions associated with protein misfolding are intermediate, soluble oligomeric aggregates rather than mature fibrils [10,12].

More recently, it was proposed that intracellular and/or extracellular protein aggregates in most neurodegenerative diseases can cross cellular membranes and directly contribute to the production of novel misfolded units and the propagation of neurodegeneration in a prion-like model [4].

## 2. Transmissible Spongiform Encephalopathies (Prion Diseases)

**Figure 1:**
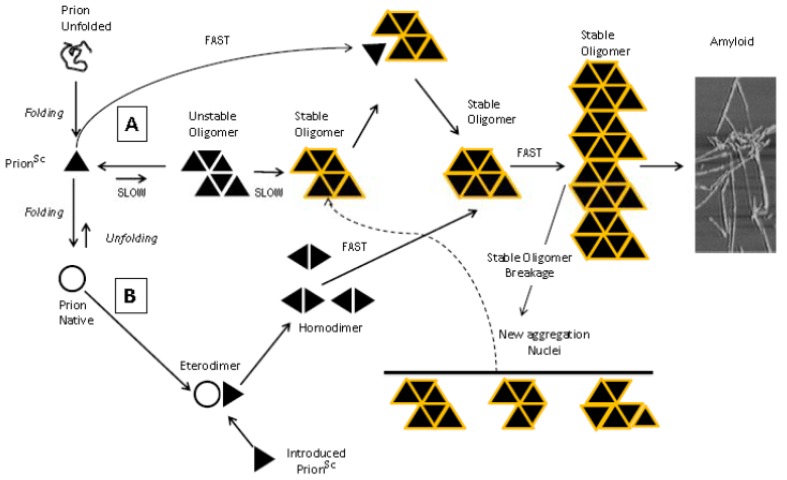
Schematic representation of the proposed mechanisms of PrP^C^ conversion into PrP^Sc^ and aggregation in amyloid fibrils. To date, two main mechanisms have been proposed to explain the auto-propagation of PrP^Sc^ from newly synthesized PrP^C^ and the final aggregation in amyloid fibrils: nucleation-polymerization (**A**) and template assisted (**B**) models. In the nucleation-polymerization model, the correct folding of newly synthesized prion peptide proceeds through distinct intermediates. Some of them are able to self-associate to form non-native oligomeric species of different sizes and structures. In the presence of stable oligomeric aggregates (illustrated by the collection of orange PrP seed triangles), a change from PrP^C^ to PrP^Sc^ is favored (**A**). In the template-assisted model (**B**), the interaction between exogenously introduced (or stochastically generated) PrP^Sc^ with endogenous PrP^C^, induces the conversion of PrP^C^ in PrP^Sc^, generating stable oligomeric aggregate. Independently from the model proposed, the growth of the stable nuclei is unlimited, generating macro-aggregate and amyloid fibrils.

TSE are fatal neurodegenerative diseases of humans and animals (Table 1) [13] that share, as a common histopathological trait, a pathognomonic triad: spongiform vacuolation of the grey matter, neuronal death, glial proliferation and, more inconstantly, amyloid deposition [14]. One of the fundamental events related to TSE pathogenesis is the refolding of a host-encoded glycoprotein, the prion protein (PrP^C^) into a protease-insensitive isoform (PrP^Sc^) that aggregates in deposits of misfolded protein. In particular, the conversion from PrP^C^ to PrP^Sc^ consists in a drastic alteration of its three-dimensional structure and, consequently, of its biochemical properties [15]. PrP^C^ secondary structure is mainly characterized by α-helices (42%), with only a small percentage of beta sheet content (3%) and the globular N-terminus non-structured. It was demonstrated that the conversion into PrP^Sc^ is driven by the transition of a large N-terminal region of PrP^C^ from random coil to β-sheet structure, which becomes predominant over the α-helices content (43% *vs*. 30%) [16]. As a result, the soluble and protease-sensitive PrP^C^ turns into the insoluble and relatively protease-resistant PrP^Sc^ [17]. According to the widely accepted “protein only” hypothesis, it was suggested that PrP^Sc^ could propagate itself by transferring its abnormal conformation on newly synthesized PrP^C^ molecules in an autocatalytic reaction [18]. Two mechanisms have been proposed to explain this conversion: (i) a nucleation-dependent polymerization process (Figure 1A), which generates a seed to recruit and stabilize the aberrant conformation of PrP^Sc^, otherwise in dynamic equilibrium with PrP^C^ [19];, and (ii) the template-assisted model (Figure 1B), which postulates an interaction between PrP^Sc^ (either exogenous or generated by stochastic conversion of PrP^C^) and endogenous PrP^C^ to induce the conformational change.

In both cases, the final event is the conversion of PrP^C^ into PrP^Sc^, a process that in normal conditions is made unlikely by high energy barrier [20].

Although rare disorders, TSEs attracted remarkable attention for their unique feature of being transmissible conditions, that can be classified, from an etiopathogenetic point of view, into sporadic, familial and acquired (infectious) forms. Acquired TSEs (caused by the accidental exposure of patients to prions), include iatrogenic CJD caused, for example, by the administration of infected post-mortem human growth hormone, the variant CJD (vCJD) derived from the consumption of meat from BSE-infected cows, and few cases of vCJD transmission by blood transfusion from affected individuals [21]. On the other hand, familial forms of prion diseases originate by pathogenic mutations within *PRNP*, the gene encoding PrP. It was proposed that these mutations favor the spontaneous conversion of PrP^C^ into PrP^Sc^ with a frequency sufficient to allow the disease to be expressed within the lifetime of the individuals [22].

The increased probability of the spontaneous PrP^Sc^ generation could be dependent on the lowered activation energy required for the transition from the normal to the pathogenic conformation, in mutants as compared to *wild type* (*wt*) PrP [23]. However, it was also suggested that the pathogenic conformation of PrP produced in familial TSE could differ from PrP^Sc^, because differences in biological characteristics were reported (for example pathogenic PrP from genetic diseases shows a much lower transmissibility than PrP^Sc^) [24,25].

Similarly to what became established for pathogenic amyloids in other neurodegenerative diseases, several studies demonstrated that intermediate oligomers of PrP^Sc^, rather than structured fibrils, are the pathogenic structures. Importantly, it was shown that, in TSE, oligomers are responsible not only for the neurodegeneration but also for the transmissibility of the disease. In this light, PrP^Sc^ oligomers attracted interest as a potential pharmacological target. In a recent study, a new class of compounds able to directly interfere with PrP^Sc^ infectivity was identified [26]. In particular, treatment with luminescent conjugated polymers (LCPs) renders PrP^Sc^, but not PrP^C^, more resistant to proteinase K (PK) proteolysis, but profoundly reduces prion infectivity through the induction of hyperstabilization, rather than destabilization, of PrP^Sc^ deposits [26].

These findings suggest that hyperstabilized mature fibrils are not correlated to prion infectivity. Moreover, a dissociation between PK resistance and infectivity of prions was highlighted, questioning the use of PK resistance as sole screening parameter to detect infective prions [26].

## 3. PrP-Derived Peptides as Models to Describe Prion Protein Aggregation Process

To study the molecular events crucial for the conversion of PrP^C^ to PrP^Sc^ and responsible of protein aggregation and neurotoxicity, high amounts of pathogenic protein are required. Since several technical issues limit the amount of PrP^Sc^ that can be purified from experimentally-infected animal brains, many studies were performed using either synthetic peptides or recombinant PrP molecules expressed in heterologous systems.

Most studies focused on the characterization of the *in vitro* misfolding/aggregation pathways of truncated forms of prion protein or PrP-derived peptides rather than the entire protein; synthetic and recombinant PrP peptides were analyzed for their structural and biological properties, including PrP106-126, PrP118-135, PrP82-146, as synthetic peptides, and PrP89-230 or PrP90-231 as recombinant mouse and human PrP isoforms [27–44].

## 4. Synthetic PrP 106-126 Peptide

The first *in vitro* model to study the pathogenic role of PrP took advantage of synthetic peptides homologous to consecutive segments of the amyloid protein purified from the brain tissue of patients with Gerstmann-Straussler-Scheinker (GSS) disease [45]. From the analysis of the biological activity of different amino acidic segments, a peptide encompassing the amino acids 106–126 of PrP sequence (PrP106-126), and able to reproduce *in vitro* several biochemical and biological properties of PrP^Sc^ (β-sheet rich structure, amyloidogenesis, and neurotoxic and gliotrophic effects), was identified [27,28,46,47]. Thus, it was proposed that this peptide contains the “death motif” internal to PrP sequence.

Circular dichroism analysis of PrP106-126, combined with *in vitro* neurotoxicity studies, revealed that the activity of this peptide is dependent on the presence of an internal hydrophobic region, as mutations in the hydrophobic residues inside this core caused inability of PrP106-126 to adopt a β-sheet conformation and to induce neurotoxicity [37].

The relevance of the 106–126 sequence in PrP biological activity was further demonstrated by the observation that this fragment contains the amyloidogenic palindrome AGAAAAGA sequence (amino acids 113 to 120) that was shown to be the most conserved region within PrP molecules in different species [48]. *In vitro*, PrP106-126 displays a β-sheet-rich structure, with a high tendency to spontaneously aggregate into amyloid fibrils [39,46,49] and partial resistance to PK proteolysis [47].

Moreover, this peptide was reported to induce apoptosis in primary cultures of hippocampal [27], cortical [50] and cerebellar neurons [36,37,51] and to exert a trophic action on glial cells [28,52,53]. Furthermore, the uptake of the peptide by neuronal and glial cells was recently demonstrated [54], suggesting that an internalized peptide may be responsible for the effects on these cells.

## 5. PrP106-126 Aggregation Is Not a Prerequisite for Its Toxic Activity

In order to elucidate whether PrP106-126 aggregation is involved in its neurotoxic activity, we generated a PrP106-126 mutant, in which two glycines within the hydrophobic palindromic core of the peptide (A_113_GAAAAGA_120_) are replaced by two alanines. We demonstrated that G_114_ and G_119_ are the main determinants for the fibrillogenesis of PrP106-126. *In silico* and experimental analysis demonstrated that the high propensity of PrP106-126 to form fibrillar aggregates was dependent on the presence of G_114_ and G_119_, while the presence of two alanines could reduce the peptide flexibility and the aggregation propensity [48]. Interestingly, this mutant PrP106-126 (PrP106-126AA) is folded in a β-sheet-rich secondary structure but, in contrast to the *wt* peptide, is completely water-soluble and does not form large aggregates or significant amounts of amyloid-like fibrils [48]. However, from a biological point of view, PrP106-126AA showed similar biological effects to the *wt* peptide, being able to induce apoptotic cell death through the activation of the p38 MAP kinase cascade [48]. These data indicate that glycines 114 and 119 in PrP106-126 sequence are major determinants required for the fibrillogenesis of the peptide, likely due to the high flexibility that they introduce in the molecular structure, but are not required for the activation of the apoptotic pathway [48]. This observation further supports the thesis that amyloid formation is not necessary for the toxic activity of PrP peptides.

Similar results were obtained using a different approach: the amidation of the *C*-terminus of PrP106-126 causes the loss of the peptide fibrillogenic activity but retains the neurotoxic activity [55].

Thus, PrP106-126 aggregation is not a prerequisite for its toxic activity.

In more recent studies, the three-dimensional structure of PrP106-126 oligomers was analyzed using solid-state nuclear magnetic resonance (NMR). A model was proposed in which these oligomers are structured as hydrated spherical assemblies, corresponding to the structure of neurotoxic Aβ oligomers in Alzheimer’s disease [56]. Thus this structure could represent a general neurotoxic conformation of amyloidogenic peptides. In detail, it was demonstrated that PrP106-126 oligomers contain the basic elements of the amyloid fibrils but are different from fibrils due to a long range disorder and local mobility [56].

## 6. Recombinant PrP-Derived Peptides

Although bacteria-synthesized recombinant PrP-derived proteins lack post-translational modifications such as the glycosylations observed in natural PrP, they share several biophysical and biological properties with PrP^C^ [57]. It is therefore generally accepted that the three-dimensional structure of recombinant PrPs is very similar or identical to the corresponding natural cellular prion proteins [58].

In several studies, PrP fragment, spanning residues 90–231, was synthesized as recombinant protein in *E. coli* to obtain a misfolded PrP conformer to better analyze the PrP structure-biological effect relationship that was not possible using small synthetic peptides [29,32,34,57,59–64]. This peptide corresponds to the protease resistant core of PrP^Sc^ that accumulates into the central nervous system (CNS) of TSE-affected patients and may represent a possible candidate responsible for the neurotoxicity of misfolded PrP [65]. *In vivo*, PrP^C^ undergoes a physiological process, generating two distinct fragments: 112-231(C1) and 90–231 (C2) [66]. However, while the C1 fragment is predominantly produced in normal CNS, a shift toward C2 generation occurs in TSE-affected brains [67,68]. Moreover, PrP90-231 contains most of the known sites in which TSE-associated point mutations occur, representing a good model also for the study of the effects of such mutations on PrP structure, aggregation and biological effects [69].

In the last few years, the three-dimensional structure of mouse, hamster, bovine and human recombinant PrPs was determined in solution by NMR spectroscopy. PrP structure from all the species was found to be very similar, being characterized by a long, flexibly disordered *N*-terminal region (residues 23–120), and a folded *C*-terminal domain (121–231) that contains three α-helices and a couple of short two-stranded, antiparallel β-sheets [70].

NMR analysis of both recombinant and brain-derived PrP confirmed that residues 23–125 form a flexible disordered tail, while residues 128–230 are organized in a globular domain, comprising three helices and two β-strands flanking helix 1 [58,71]. The unstructured portion of PrP, between residues 90 and 120, undergoes profound rearrangements in PrP^Sc^ [72], and spontaneously forms amyloid *in vitro* when synthesized as an isolated peptide [45,73], indicating that this region is essential for PrP^C^ refolding and misfolding and, possibly, PrP^Sc^ aggregation.

Consistent with this view, PrP fragments deleted in the regions encompassing residues 109–112 or 114–121 are refractory to the conformational conversion induced by PrP^Sc^, and have a trans-dominant inhibitory effect on the conversion of *wt* PrP into the pathological isoform [74,75]. Interestingly, among the three PrP α-helices, synthesized as isolated peptides, only helix-3 was endowed with autonomous self-structuration, due to the presence of a *N*-capping box and partially for the formation of an ionic bond between E_200_ and K_204_ [76]. Moreover, the helix 3 structure was lost when the GSS related mutation D202N was introduced, providing a potential structural mechanism for the PrP^Sc^ generation in this condition [76].

## 7. Determinants of Structural Misfolding of Recombinant PrP Fragments

The first report of the use of a recombinant PrP peptide to study the molecular rearrangements responsible of PrP^Sc^ formation and to study its fibrillogenic and neurotoxic effects was provided using human PrP91-231 expressed in *E. coli* [57]. In particular, the peptide was purified as a highly soluble monomer, rich in α-helical structures and showing a single intact disulfide bridge. Reduction of the disulfide bond and lowering the pH to 4.0 generated a different PrP91-231 conformation, characterized by high β-sheet content and an increased tendency to form amyloid aggregates, through the stabilization of the intermolecular interactions among β-structures. Importantly, the switch from α to β conformations was reversible, when the reduced β form was exposed to higher pH (8.0), although the rate of the reverse conversion was extremely slow [57]. A variety of additional factors able to induce the structural conversion of recombinant PrP molecules have been successfully established in cell-free systems, including: (i) incubation in high salt concentration or in the presence of chaotropic agents [29,77,78]; (ii) controlled thermal denaturation [34]; (iii) high pressure [79], and (iv) brain purified PrP^Sc^-seeds [80]. Baskakov *et al*., demonstrated that different pathways are activated to generate PrP oligomers or amyloid fibrils. In particular, the differential refolding of PrP90-231 from α-monomers to β-oligomers or amyloid fibrils can be induced, modifying denaturation conditions (partial denaturation in acidic pH induce oligomer formation, while strong denaturation at neutral pH and continuous shaking generate amyloid fibrils) [29,77].

**Figure 2:**
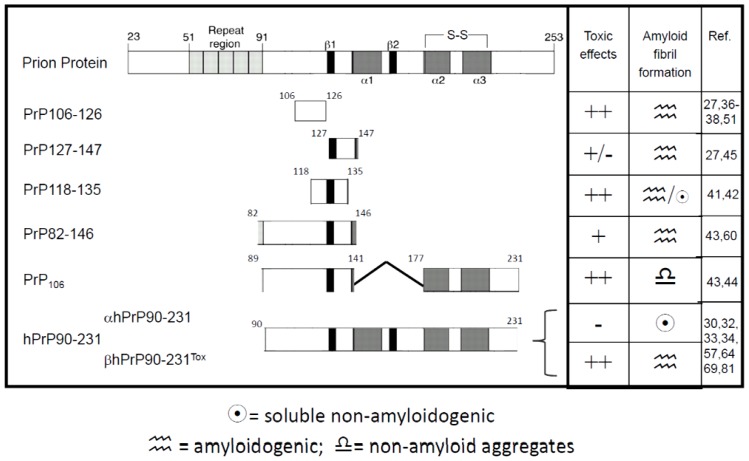
Schematic representation of the most commonly used synthetic and recombinant PrP peptides, and their main biophysical and biological characteristics.

In Figure 2 is a summary of the most commonly used PrP-derived peptide and their main characteristic regarding aggregation and *in vitro* toxicity [27,30,32–38,41–45,51,57,60,64,69,81].

In our studies, recombinant human PrP90-231 peptide was isolated as an isoform that reproduced PrP^C^-like features [81]. This peptide was purified as a native soluble protein, adopting a protocol that avoids the requirement of denaturation-renaturation cycles to dissolve bacteria inclusion bodies; hPrP90-231 is largely structured in α-helix and retains an intact intra-molecular disulphide bridge. By limited thermal denaturation (1 hr at 53 °C), hPrP90-231 was converted into a β-sheet rich, insoluble, aggregation-prone, hydrophobic peptide (all of these representing features identified in PrP^Sc^) [34]. Moreover, in this conformation, but not in its native α-helix structure, hPrP90-231 induces cell death through caspase-dependent apoptosis, mimicking the effects of PrP^Sc^ purified from infected Hamster brain [34,82,83].

Because hPrP90-231 cytotoxicity is completely dependent on the three-dimensional structure [59,61,82], the peptide rich in β-sheet structures has been named hPrP^Tox^ [84].

## 8. Recombinant Prion Peptides Infectivity

Although aggregated isoforms of recombinant peptides have been characterized as PrP^Sc^-like molecules, the reconstitution of infectivity is still difficult to achieve *in vitro* [85]. The generation of self-propagating amyloid fibrils of recombinant mouse PrP89-230 was demonstrated by continuously shaking in slightly acidic solution in the presence of denaturating agents (2–4 M urea) [29]. These fibrils, generated by MoPrP89-230, when intracerebrally inoculated into transgenic mice expressing the same sequence (MoPrP89-230) are able to induce a prion-like disease at the second *in vivo* passage [86].

More recently, Fei Wang *et al*., reported the generation of a murine recombinant PrP (rPrP23-230) with the attributes of the pathogenic PrP isoform able to infect *wt* mice after intracerebral injection. Interestingly, in this model, incubation with a synthetic anionic phospholipid POPG (1-palmitoyl-2-oleoylphosphatidylglycerol) and RNA isolated from normal mouse liver were instrumental in the induction of prion infection, thus proposing that lipids play an essential role in PrP conversion into an infective unit [87].

## 9. Mechanisms of Aggregation and Neurotoxicity of Recombinant Prion Peptides

The observation that in most models, *in vitro*-generated fibrils are non-toxic species [88], supports the hypothesis that soluble oligomeric intermediates rather than structured amyloid are the causative agents of neuronal cell death in TSE, as it was already proposed in other neurodegenerative disorders associated with protein misfolding and aggregation [89,90].

In this regard, β-sheet enriched oligomers have been detected on the pathway of PrP aggregation *in vivo* [91,92] and *in vitro* [77,78,93,94].

Also in our studies, pre-fibrillar, monomeric or small oligomeric conformers of hPrP^Tox^, rather than soluble or fibrillar large aggregates, represent the neurotoxic species [95]. In particular, we monitored the time-course of hPrP90-231 aggregation by electron microscopy, Congo red binding, and evaluating the loss of energy of the transmitted light (apparent absorbance) at 380 nm. We reported that mild thermal denaturation of hPrP90-231 (1 hr at 53 °C), while sufficient to generate hPrP^Tox^, does not induce the formation of large aggregates or structured fibrils [95]. In fact, only after very prolonged incubation at 37 °C, did hPrP90-231 generate amyloid-like fibrils that however, lose their toxic potential [95].

Similarly, Simoneau *et al.*, demonstrated that β-PrP oligomers derived from ovine PrP23-234, are the major neurotoxic species *in vitro* and *in vivo*, confirming that this aggregation isoform is likely to be the PrP structure responsible of the development of TSE-linked neurodegeneration [96]. Using recombinant ovine PrP fragments [ovPrP (25–234)], corresponding to scrapie-resistant and susceptible genotypes (Ala136/Arg154/Gln171, ARR and Ala136/Arg154/Arg171, ARQ, respectively), it was confirmed that, in contrast to what observed for the transmission of TSE, PrP-ARR was much more toxic for neurons in culture than PrP-ARQ, due to the lower structural stability that reduced the ability to form large aggregates and fibrils [97].

From a molecular point of view, hPrP^Tox^ small oligomers induce neurotoxicity after neuron internalization and accumulation into the endolysosomal compartment. These aggregates cause lysosomal damage, proteolytic enzyme leakage and activation of caspase-dependent apoptosis [61,95]. In these studies, we demonstrated that fibrillar hPrP90-231 isoform does not accumulate into acidic vesicles, suggesting that the inability of hPrP^Tox^ fibrillar or large aggregates to be internalized abolishes its neurotoxic potential.

**Figure 3:**
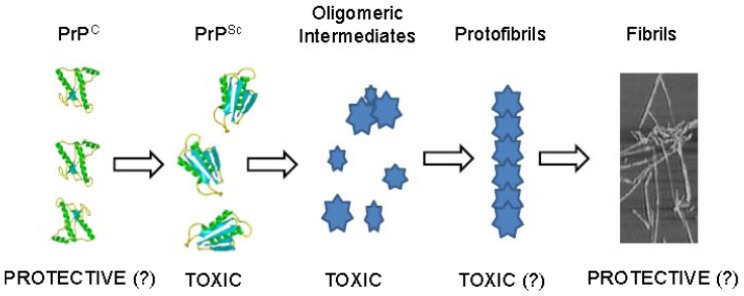
Biological significance of intermediate products during prion aggregation process. Schematic representation of prion aggregation steps. Monomeric PrP^Sc^, small oligomers or PrP pre-fibrillar structures are believed to be the causative agents of prion-dependent neuronal death. Recent experimental evidence suggests a possible cellular protective role for PrP^C^ and fibrils.

All this experimental evidence supports the notion that prion disease neurotoxicity is mediated by misfolded protein oligomers that constitute the major trigger of the pro-apoptotic process, rather than large fibrillar aggregates (Figure 3).

Some relevant aggregation studies also utilized recombinant full-length PrP (a.a. 23–231) [78,98], however, aggregation properties of this construct have not been analyzed as extensively as those of the truncated forms, perhaps because the *C*-terminal portion is the protease-resistant portion of PrP^Sc^ and it was assumed that this fragment retains most of the relevant information about PrP aggregation and toxicity [99]. However, using recombinant full-length PrP, it was recently shown that beside β-oligomers, some monomeric and α-helix structured PrP species are also able to induce neurotoxic effects *in vivo* and *in vitro* [100]. This evidence belies the assertion that prion neuronal damage is only or predominantly linked to the toxicity of β-sheet PrP oligomers, opening up a new chapter in the understanding of prion-induced neurodegeneration, that still needs to be explored.

In other studies, PrP fragments with *N*-terminal deletions were shown to form atypical conformations of prion aggregates. Lawson *et al*., introduced deletions into the flexible *N*-terminal region of hamster PrP (residues 34–124) and investigated the effect of this region in conformation of the protease-resistant form of the host-encoded prion protein (PrP-res). PrP-res formation, generated *in vitro* in a cell-free conversion assay, was significantly reduced by deletion of residues 34–94 relative to full-length hamster PrP [101,102]. It was observed that the flexible *N*-terminal tail of PrP influenced the interactions required for either generating or disrupting PrP-res formation, suggesting that this region may influence TSE pathogenesis and transmission [101,102].

Insight into the mechanisms of PrP aggregation can be obtained by examining the dependence of its rate of polymerization on protein concentration. The aggregation of numerous proteins involved in neurodegenerative diseases has been proposed to proceed by a nucleation-dependent mechanism, representing the most widely accepted model for this type of process [103–105]. In this model, the formation of the highest energy species, the thermodynamic nucleus, from monomeric units, is rate-limiting for the formation of larger aggregates. The rate of aggregation has a high-order dependence on the concentration of monomeric protein, a mechanism that has been used to explain the late onset of sporadic forms of Alzheimer’s and prion diseases [106]. Recently, starting from the evidence that the aggregation is triggered by a seeding mechanism, in which PrP oligomers are able to catalyze polymerization of further prion proteins into larger aggregates (see Figure 1), Hesketh *et al*., analyzed the substrate interaction with oligomeric species, generated from recombinant PrP. They demonstrated that different domains within PrP are required to generate the seed as compared to those necessary to induce further aggregation [107].

## 10. Misfolding/Aggregation Pathways of E200K or D202N Disease-Related Mutations of the Human Prion Protein

Approximately 15% of human prion diseases have a pattern of autosomal dominant inheritance, and are linked to mutations in the PrP gene encoding (*PRNP*). More than 30 pathogenic mutations in human *PRNP* have been identified (Table 2) [108]. The exact role of these mutations in TSE pathogenesis has not been completely elucidated and significant differences with the infective PrP^Sc^ isoform were observed. For example, genetic TSEs show a very low rate of transmissibility [24,25]. In fact, by expression of mutant PrP molecules in several cell types and tissues, it was reported that E200K mutation, observed in familial CJD patients, does not automatically convey the properties of PrP^Sc^ to new synthesized PrP molecules. This conversion process occurs mainly in affected brains, suggesting the presence of a tissue-specific or age-dependent factor, accordingly with the late onset nature of inherited CJD [109]. It was initially proposed that PrP mutations may facilitate the spontaneous generation of PrP^Sc^, destabilizing the native structure of PrP^C^. Zhang *et al*., studying E200K variant of human PrP, assessed that the backbone tertiary structure of this mutant PrP is nearly identical to that reported for the *wt* human PrP. The only major consequence induced by the mutation on PrP structure is the perturbation of surface electrostatic potential, suggesting that not all hereditary TSEs can be rationalized through a common mechanism based on thermodynamic destabilization of PrP^C^ [110]. Conversely, Hasegawa *et al*., performed *ab initio* fragment molecular orbital calculations for the *wt* human PrP and the E200K variant, modeled under neutral and mild acidic conditions. This substitution markedly altered the intra-molecular interactions in the PrP, suggesting that the local structural instabilities induced by the E200K mutation might cause initial denaturation of the PrP and its subsequent conversion to a pathogenic isoform [111].

Recent evidence suggests that *PRPN* mutations may not only influence PrP^C^ stability, but also its processing and/or its protein-to-protein interactions, such that aggregation can occur and disease develops [112,113].

Notwithstanding all these data, the cellular mechanisms by which PrP mutations cause the formation of neurotoxic species are still largely unknown. Recombinant D202N and E200K mutants of hPrP90-231 were synthesized to analyze the influence of these mutations on the biochemical and biological properties of the protein [114]. hPrP90-231 *wt* and the D202N mutant were non-toxic in their native conformation, but caused cell death only after misfolding induced by controlled thermal denaturation. Conversely, the introduction of the E200K mutation produced a highly toxic peptide already in its native structure, suggesting that E200K mutation *per se* favors the acquisition of a toxic conformation, at least within the truncated 90–231 sequence [114].

hPrP90-231 E200K mutant showed a high propensity to aggregate, as opposed to hPrP90-231 *wt* and D202N mutant that adopt this feature only after thermal denaturation. Interestingly, resistance to PK proteolysis was also identified in native hPrP90-231 E200K. Similar results were reported using the full length PrP bearing E200K mutation in which PK resistance was identified as an intrinsic feature [115]. These data demonstrate that most of the structural features of *wt* and mutant full length PrP molecules are indeed retained in the recombinant 90–231 fragments.

It was proposed that the toxicity of *wt* hPrP90-231 oligomers or of E200K and D202N mutants can be effectively explained by a common mechanism, corresponding to an increase in hydrophobic amino acid exposure that is responsible for both aggregation profiles and toxic activity, likely through an increased interaction with neuronal membranes and cell internalization [114]. Observed hydrophobicity changes in hPrP90-231 in the presence of E200K mutation well fit with other reports [111] in which it was demonstrated, by fragment molecular orbital calculations, that E200K mutation causes a drastic change in PrP intra-molecular interactions, leading to the structural rearrangement of PrP altering its local structural stability.

## 11. Effect of Soil Composition on Recombinant PrP Aggregation and Neurotoxicity

Soil composition undoubtedly affects retention, aggregation and decomposition of infectious and toxic bio-macromolecules as well as that of nucleic acids, and it is well known that easily decomposable substances can be stabilized by interaction with soil [116].

Prion interactions with soil components may play an important role in the transmission of TSE, and it is a matter of fact that prions derived from infected animals can contaminate soil.

Agricultural spreading of infected organic manures may have contributed to prion dissemination during the BSE epidemic, before appropriate safety regulations were established [117]. The relevance of soil composition in TSE spreading was demonstrated by the requirement of the presence of Cu^++^, Mn^++^ and other trace metals in soil for the transmission of the disease [118,119].

Prions enter the environment in complex, competitive matrices, such as urine, feces, saliva, blood, and birthing matter, as well as tissue from mortalities [120,121]. Environmental persistence of prions in soil was demonstrated by intra-cerebral bioassays, after mixing infected cerebral tissue with soil packed into perforated Petri dishes that were then embedded within soil-containing pots, and buried in a garden for three years [122]. Dissolved ions present in solution can interact with both mineral surfaces and proteins to impact soil adsorption capacity and the conformation of adsorbed proteins [123]. Saunders *et al*., evaluated differences in prion adsorption and replication efficiency as a function of adsorption solution and measured desorption and replication of soil-bound prions over time periods of up to one year. They demonstrated that after binding to soil components, prions remain a risk of disease transmission for long time [123]. Moreover, they observed that PrP^Sc^ bound to soils with high clay or organic component contents exhibit a reduced ability to initiate conversion of PrP^C^ into pathogenic conformer, while after binding to sand surfaces PrP^Sc^ exhibits no such reduction [124]. The 263K scrapie agent can persist in soil for at least 29 months and the oral administration of contaminated soil extracts is able to transmit disease to Syrian hamsters [125]. Moreover, prions bound to soil mineral montmorillonite (Mte) significantly enhance disease penetrance and reduce the incubation period as compared to unbound molecules [126]. This effect was observed in two out of three soils tested, and this discrepancy was attributed to the different organic component present in these soils that may affect the access of PrP^TSE^ to sorption sites on mineral surfaces [126]. These data clearly indicate that different soil component may contribute to the environmental spread of TSEs by increasing or reducing the transmissibility of small amounts of the prion infectious agent dispersed in the environment. Epidemiological studies showed that scrapie and CWD could be horizontally transmitted to animals grazing in pastures or paddocks contaminated by infected carcasses, feces and urine excreted by infected animals, or infected placenta remaining on the ground after young are born [117,127]. The interaction of hPrP90-231 with humic substances (HS) was analyzed at molecular level to verify whether different soil components could affect the generation of hPrP^Tox^ [128]. HS are refractory components of natural organic matter in soil, sediments and waters, widely diffused in all climatic environments and naturally accumulated in soil under cool temperate climates. They are classified as humic acids (HA), which are soluble only in alkaline solutions, and fulvic acids (FA), which are soluble in both alkaline and acid solutions.

*In silico* docking calculations were performed to identify interacting sites on hPrP90-231 with two model structures of HS: a model HA and a model of Swannee River FA, showing a significant interaction at several different positions on the surface of hPrP90-231 [128] as also observed using the mouse PrP89-231 [129]. Both HA and FA significantly bind to hPrP90-231 at several amino acid residues located in a region (α-helix A and β-sheet S1) relevant for the conversion of PrP^C^ to PrP^Sc^, possibly interfering with its conformational change, aggregation and neurotoxic activity. Moreover, the binding of HS to hPrP90-231 inhibited the acquisition of several PrP^Sc^-like characteristics, including PK resistance, but induced the formation of large protein aggregates that prevent the internalization of hPrP^Tox^ conformers into living cells, likely removing their toxicity [128]. This evidence concerning the effects of HS on the conversion of hPrP90-231 in hPrP^Tox^ supports the hypothesis that soil composition, when rich in humic substances, may affect prion infectivity and toxicity, thus explaining why TSE transmission in wild animals is restricted to specific geographic areas [128].

## 12. Conclusions

The understanding of the molecular mechanisms involved in the PrP^C^ conversion into PrP^Sc^ and how this pathogenic protein may aggregate and cause neuronal cell death is an important target for biomedical and, possibly, pharmacological research.

Synthetic and recombinant prion fragments represent suitable tools to study the cellular and molecular effects of PrP^Sc^ *in vitro.* In particular, the production of large recombinant *N*-truncated PrP fragments allowed the determination of a precise structure-effect relationship.

In this review we summarized the main experimental evidence regarding PrP aggregation determinants obtained using different synthetic and recombinant PrP peptides. To date, although progress towards the detailed comprehension of PrP biology and TSE pathogenesis is significantly advanced, possible pharmacological approaches, able to at least slow down the progression of the disease, have not been identified. Indeed, TSEs are still inevitably fatal, and there is no evidence that any patients or experimental animals suffering from a clinically manifest TSE have ever been cured. However, we believe that the identification of the mechanisms by which PrP can be converted in infective and/or neurotoxic entities may provide valuable information for the identification of novel potential pharmacological approaches for TSEs and other neurodegenerative diseases. In this context, PrP recombinant peptides may be a precious tool to delve deeper into the molecular determinants of the potential interaction of prion-like molecules with potential new drugs, as already demonstrated in recent neurotoxicity studies [64].

## Figures and Tables

**Table 1:** Prion diseases.

Disease	Affected Species
Kuru	***Human***
Creutzfeldt-Jakob disease (CJD)	
sporadic Creutzfeldt-Jakob disease (sCJD)	
iatrogenic Creutzfeldt-Jakob disease (iCJD)	
variant Creutzfeldt-Jakob disease (vCJD)	
familial Creutzfeldt-Jakob disease (fCJD)	
Gerstmann-Sträussler-Scheinker syndrome (GSS)	
Fatal familial insomnia (FFI)	

Scrapie	***sheep*,** ***goat cattle elk*,** ***mule deer*,** ***moose cat***
Bovine spongiform encephalopathy (BSE)	
Chronic wasting disease (CWD)	
Feline spongiform encephalopathy (FSE)	

**Table 2:** Representative point mutations responsible of familial prion diseases.

Prion Protein Mutation	Prion Disease
**P102L, P105L, A117V, G131V, F198S, D202N, Q217R, M232T**	Gerstmann-Straussler-Scheinker disease (GSS)
**D178N + (variant Val 129) V180N, M232R, T183A, E200K, R208H, V210I, M232R**	Creutzfeldt-Jakob disease (CJD)
**D178N + (variant Met 129)**	Fatal familial insomnia (FFI)
